# Improved tools and strategies for the prevention and control of arboviral diseases: A research-to-policy forum

**DOI:** 10.1371/journal.pntd.0005967

**Published:** 2018-02-01

**Authors:** Piero Olliaro, Florence Fouque, Axel Kroeger, Leigh Bowman, Raman Velayudhan, Ana Carolina Santelli, Diego Garcia, Ronald Skewes Ramm, Lokman H. Sulaiman, Gustavo Sanchez Tejeda, Fabiàn Correa Morales, Ernesto Gozzer, César Basso Garrido, Luong Chan Quang, Gamaliel Gutierrez, Zaida E. Yadon, Silvia Runge-Ranzinger

**Affiliations:** 1 UNICEF/UNDP/World Bank/WHO Special Programme for Research and Training in Tropical Diseases (TDR), World Health Organization, Geneva, Switzerland; 2 Global Health Department, Centre for Medicine and Society/Anthropology, Freiburg University, Freiburg im Breisgau, Germany; 3 Department of Vector Biology, Liverpool School of Tropical Medicine, Liverpool, United Kingdom; 4 Department of Control of Neglected Tropical Diseases (WHO/NTD), World Health Organization, Geneva, Switzerland; 5 Dengue Control Program, Ministry of Health, Brasilia, Brazil; 6 Department of Communicable Diseases, Ministry of Health, Bogota, Colombia; 7 Program for the Prevention and Control of Dengue, Ministry of Health, Santo Domingo, Dominican Republic; 8 Department of Public Health, Ministry of Health, Kuala Lumpur, Malaysia; 9 Centro Nacional de Programas Preventivos y Control de Enfermedades (CENAPRECE), Ministry of Health, Mexico City, Mexico; 10 Universidad Peruana Cayetano Heredia, Lima, Peru; 11 Facultad de Agronomia, Universidad de la República, Montevideo, Uruguay; 12 Department for Disease Control and Prevention, Pasteur Institute, Ho Chi Minh City, Vietnam; 13 PAHO/AMRO, World Health Organization, Washington, DC, United States of America; 14 PAHO/AMRO, World Health Organization, Rio de Janeiro, Brazil; 15 Institute of Public Health, University of Heidelberg, Heidelberg, Germany; Faculty of Science, Mahidol University, THAILAND

## Abstract

**Background:**

Research has been conducted on interventions to control dengue transmission and respond to outbreaks. A summary of the available evidence will help inform disease control policy decisions and research directions, both for dengue and, more broadly, for all *Aedes*-borne arboviral diseases.

**Method:**

A research-to-policy forum was convened by TDR, the Special Programme for Research and Training in Tropical Diseases, with researchers and representatives from ministries of health, in order to review research findings and discuss their implications for policy and research.

**Results:**

The participants reviewed findings of research supported by TDR and others.

**Surveillance and early outbreak warning**. Systematic reviews and country studies identify the critical characteristics that an alert system should have to document trends reliably and trigger timely responses (i.e., early enough to prevent the epidemic spread of the virus) to dengue outbreaks. A range of variables that, according to the literature, either indicate risk of forthcoming dengue transmission or predict dengue outbreaks were tested and some of them could be successfully applied in an Early Warning and Response System (EWARS).

**Entomological surveillance and vector management.** A summary of the published literature shows that controlling *Aedes* vectors requires complex interventions and points to the need for more rigorous, standardised study designs, with disease reduction as the primary outcome to be measured. House screening and targeted vector interventions are promising vector management approaches. Sampling vector populations, both for surveillance purposes and evaluation of control activities, is usually conducted in an unsystematic way, limiting the potentials of entomological surveillance for outbreak prediction.

Combining outbreak alert and improved approaches of vector management will help to overcome the present uncertainties about major risk groups or areas where outbreak response should be initiated and where resources for vector management should be allocated during the interepidemic period.

**Conclusions:**

The Forum concluded that the evidence collected can inform policy decisions, but also that important research gaps have yet to be filled.

## Background

As dengue (DENV), chikungunya (CHIKV), and Zika viruses (ZIKV) continue to spread worldwide, there is an ever-increasing need to develop and apply cost-effective, evidence-based approaches to identify and respond to arboviral disease outbreaks. Outbreak response should be timely (i.e., early enough to prevent the epidemic spread of the virus), coordinated among multiple stakeholders, and should make use of existing vector control interventions against *Aedes* populations—the principal vector responsible for the transmission of these viruses.

**Fig 1:**
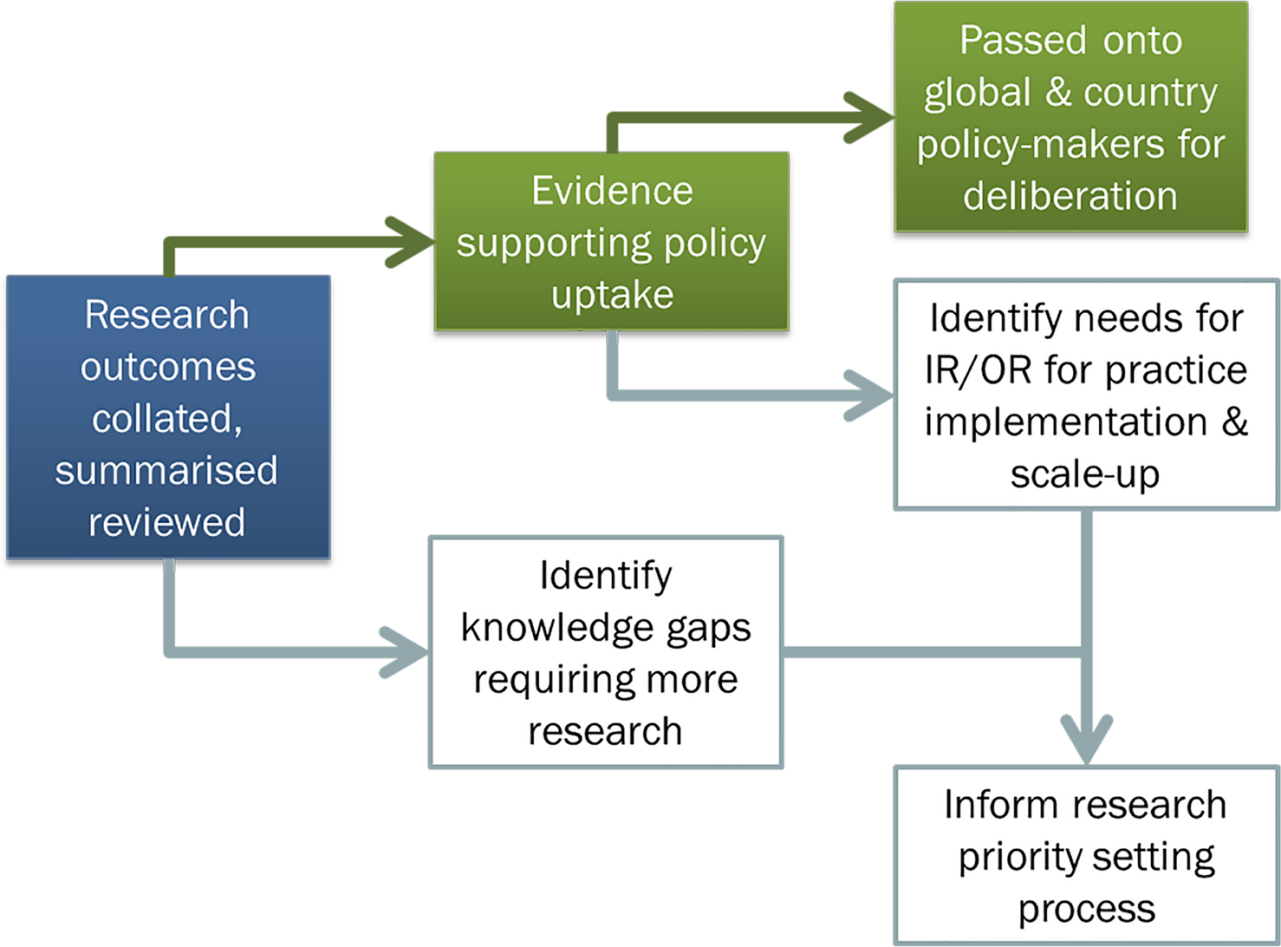
Evidence generation to inform policy decisions and further research requirements. IR, Implementation Research; OR, Operational Research.

Research has been conducted on interventions to control dengue transmission and outbreak preparedness, including work supported and coordinated by TDR, the Special Programme for Research and Training in Tropical Diseases. A substantial body of information has been generated over 13 years, and a summary of the available evidence would presently be helpful both to the research and disease control communities. It also remains crucial to establish the various capacity-strengthening needs that countries require to test, deploy, and monitor interventions. Indeed, such information could be used to inform policy decisions as well as identify knowledge gaps (see Fig 1).

Accordingly, this article provides the necessary summary complemented by valuable input from a research-to-policy forum held in August 2016 in Geneva, Switzerland. The forum reviewed the output of research on tools for early dengue outbreak detection and response, as well as improved approaches to dengue vector control that have been developed and tested by TDR with its partner research institutions and country control programmes, against the background of the broader research context.

Specifically, this paper reviews the output of research on (1) outbreak preparedness of dengue epidemics and (2) innovative vector control interventions to control *Aedes*–borne disease transmission.

## Summary of findings of TDR-supported research on dengue outbreak detection and innovative vector interventions

### Outbreak preparedness of dengue epidemics

#### Effective dengue, chikungunya and Zika surveillance

Routine reporting of notifiable diseases in national disease surveillance systems is the backbone of epidemiological information; it is used to monitor the spatial and temporal distribution of different clinical expressions of diseases, to determine the risk and priority areas for interventions, and to trigger outbreak alerts.

Dengue-endemic country surveillance and response systems were systematically reviewed to identify what corrective actions should be undertaken and how countries should be supported. Dengue surveillance was found to suffer from significant delays and marked underreporting, especially for nonhospitalized dengue cases [1]. The comparative analysis of national dengue contingency plans [2] revealed weak overall outbreak governance due to poor clarity of stakeholder roles, weak surveillance systems, inadequate use of routinely generated data and additional alerts in tandem, absence of outbreak definition, and absence of structured early-response mechanisms.

Systematic reviews and country studies highlighted the critical characteristics of an efficient alert system to trigger responses: it should be sensitive to predict or detect outbreaks in a timely manner; specific to avoid unnecessary false alerts; and timely to trigger early response [3]. In order for systems to meet these requirements, they should make use of a simplified and standardized case classification [4], be supported by laboratories using standardized and quality-controlled assays, include active/enhanced/syndromic surveillance, and either incorporate additional alarm signals or increase data quality and/or timeliness [3].

Laboratory diagnosis of dengue is currently done either by detecting the virus or its components (viral RNA by PCR, or antigens like NS1) or by detecting immune response by serological tests. A “confirmed case” requires either virus isolation, RNA detection, antigen detection, seroconversion for IgM, or a 4-fold rise in IgG titres; IgM positivity is considered highly suggestive. The timing of testing is critical, considering that both viraemia and NS1 are confined to the first week of illness, and IgM production is transient (lasting 5–6 months), while IgG lasts longer [5]. Confirming dengue diagnosis is a clear challenge for countries, both at point-of-care and at referral facilities. However, the combination of PCR or NS1 ELISA plus IgM ELISA appears to accurately identify >90% of primary and secondary dengue cases from a single serum specimen collected during the first 10 days of illness [6,7]. While operationally and financially demanding, laboratory confirmation is important, both for clinical management and outbreak identification: it increases the specificity of the information captured by the surveillance system, contributes to syndromic surveillance (e.g., increased numbers of laboratory requests), and may generate serotype- or genotype-specific data as a potential additional outbreak alarm signal.

Syndromic surveillance [8] (developed as an additional, often context-specific tool for early outbreak alert) is not limited only to clinical syndromes but may include increased numbers of school absenteeism, increased laboratory requests—as described above—or an increased proportion of positive laboratory results in the inter-epidemic period. These alarm signals can be then integrated into a risk assessment tool (see below). Indeed, enhanced surveillance should aim to combine tools that complement routine reporting, not to replace it [9].

One question is whether and how what we know about dengue outbreak detection can be applied to Zika and chikungunya. For dengue, we have years of historical incident case data and can construct an “endemic channel.” We do not have this information for the other two, which have so far occurred in naïve populations; therefore, syndromic and sentinel-based approaches seem the best option at this point in time until comprehensive, historic datasets become available.

#### Outbreak preparedness and response

**Fig 2:**
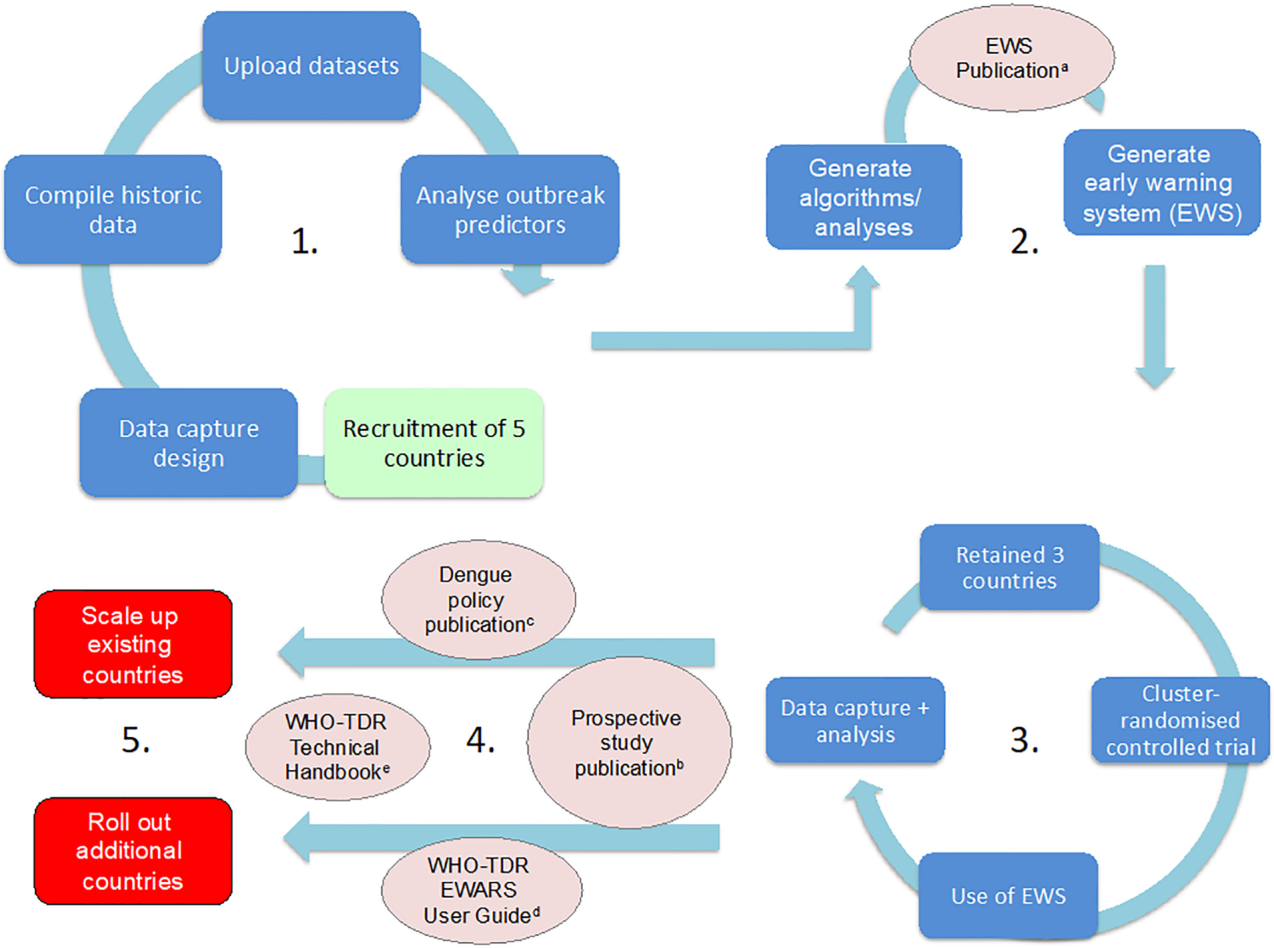
The process: From research to operational implementation. (1) Retrospective analysis of alarm indicators for dengue outbreaks. (2) Use of algorithms to generate prospective early warning system (EWS). (3) Prospective randomised controlled trial of early warning and response system (EWARS). (4) Associated EWARS publications (5) Scale up of EWARS. ^a^ Bowman et al.2016; ^b^ Cluster-randomized controlled trial for dengue early warning systems *(in-prep)*; ^c^ Runge-Ranzinger et al. 2016; ^d^ WHO-TDR. Early Warning and Response System (EWARS) for Dengue Outbreaks: Operational Guide *(in-press);* ^e^ WHO-TDR. Technical handbook. 2016.

A range of variables that either indicate risk of forthcoming dengue transmission or predict dengue outbreaks have been suggested throughout the literature [10,11,12]. The combination of these variables, or alarm indicators, for use in early warning systems is the next natural step. An Early Warning and Response System (EWARS) has since been developed following a period of retrospective country dataset analysis [13], modelling, and prospective field evaluation (Fig 2). This recent research has also included the development of a “staged” response system, which gradually introduces greater intervention resources in response to increasing certainty of forthcoming outbreaks. The outbreak warning system (EWS)—a combination of statistical (STATA) and database management (Microsoft Excel) software—will be freely available via the publication *Early Warning and Response System (EWARS) for Dengue Outbreak*: *Operational Guide* (WHO-TDR)[14]. These tools and materials will help build capacity in dengue-endemic settings.

In addition, a technical handbook (model contingency plan) has been developed. It is intended to serve as a framework, incorporating all under the aspects of evidence described in “Outbreak preparedness of dengue epidemics” above in order to support and guide the national contingency-planning process [15]. Details of this framework are summarized and published in a separate publication [9].

Following up the above-mentioned evidence, two additional areas of future development have emerged: (1) the ability to combine qualitative and quantitative variables into a broad-ranging early warning system, and (2) spatial risk-mapping tools that help to identify smaller spatial areas for fine-scale interventions.

### Improved vector interventions to control *Aedes*-borne disease transmission

#### Systematic reviews on dengue vector management

A systematic review and meta-analysis [16] evaluated the evidence of the effectiveness of vector control interventions in (a) reducing vector indices and (b) preventing dengue transmission. The searches covered all major indexing databases throughout the period 1980–January 2013. The primary outcome was dengue incidence with secondary outcomes comprising a number of *Aedes* indices (e.g., Breteau Index, House Index, Container Index, mosquito adults per person, pupae per person); analyses were stratified by study design, intervention, and measures of effect and outcome. The main findings of the meta-analysis were: (1) moderate evidence that house screening can reduce vector abundance and emerging evidence that it reduces dengue transmission; (2) strong evidence that community-based campaigns can impact vector abundance, with emerging evidence for impact on transmission; and (3) no robust studies on the impact of fogging on transmission, with only one study showing an impact on *Aedes albopticus*. The only evidence to-date of effectiveness in preventing dengue transmission is in the form of house screening; the meta-analysis demonstrated a significant reduction in the odds of dengue incidence among households with screens (three studies, pooled odds ratio: 0.22 [95% confidence interval: 0.05, 0.93]) compared to unscreened homes. These findings are corroborated by new investigations of screening in Mexico, which have shown window and door screens to be a popular and widely-adopted intervention that can significantly reduce domestic infestations of *A*. *aegypti* [17,18]

When data were not appropriate for meta-analysis, single interventions have been described in systematic reviews elsewhere that address peridomestic space spraying [19], temephos [20], *Bacillus thuringiensis israelensis* [21], copepods [22], and larvivorous fish [23]. Further work has been completed on the effectiveness of pyriproxyfen [24] and work on indoor residual house spraying (IRS), including targeted indoor spraying, is under way. In summary, these results indicate that as single, standalone interventions, these approaches are efficient; however, little evidence exists with respect to reduction of disease incidence.

A summary of the published literature to-date can be described as follows: (1) Vector control can be effective against *Aedes* populations; (2) Effectiveness against transmission has been reported in a minority of robust study designs—implementation remains an issue; (3) Single interventions may be largely ineffective with respect to disease reduction in the community; (4) Complex interventions have proven very effective at reducing vector abundance as part of community-based campaigns; (5) Early vector control implementation prior to outbreaks would likely have greater impact at mitigating dengue cases; (6) Methodologically rigorous studies are required to contribute further evidence towards effective vector control strategies.

#### Systematic review on entomological surveillance

A systematic literature review [25] examined the published evidence investigating associations between vector indices and dengue cases. After assessment of the epidemiological study designs, all but three of the 18 studies were classified as methodologically weak. Heterogeneity among spatial/temporal sampling and analyses was high, perhaps demonstrating an absence of standardization for conducting such research. Of the 13 studies that investigated associations between vector indices and dengue cases, 4 reported positive correlations, 4 found no correlation, and 5 reported ambiguous or unreliable associations. Of the 7 studies that measured the Breteau Index, 6 reported dengue transmission at levels below the widely used threshold of 5.

Sampling vector populations, both for surveillance purposes and evaluation of control activities, is conducted annually worldwide. Further evidence of the relationship between vector indices and dengue transmission is necessary to better understand the impact of control activities on dengue incidence. Also, the role of asymptomatic individuals in virus transmission requires further analysis.

#### Research on house screening and targeted vector interventions

The research programme labelled “eco-bio-social research” in five Asian and five Latin American countries has, in its first phase, identified the complexity of determinants for vector breeding [26,27,28] and, in its second phase, identified innovative vector control interventions delivered through partnership models including vector control services, communities, and others. The interventions included protection from adult vectors through insecticide-treated window curtains or screens and/or targeting productive oviposition sites (i.e., in those container types which produce more than 70% of all *Aedes* pupae) [29,30,31]. While the intervention packages including treated window curtains were all able to reduce vector densities significantly, reduction of dengue incidence could not be used as an end-point measure due to relatively small intervention clusters. In settlements with rather compact houses (i.e., houses with few or no openings additional to windows and doors) [32,33] the effect of fixed window screens with insecticide-treated netting materials seemed to have an even stronger and longer–lasting effect, and are well accepted and affordable if certain cost-saving strategies are introduced [18,34,35]. Cost savings are expected to occur with the massive industrial production of window screens, the increased demand by the middle and upper classes, and by the use of cheaper materials (wooden rather than aluminum frames).

### Bringing outbreak alert and integrated vector management together: Research needs and expected results

**Fig 3:**
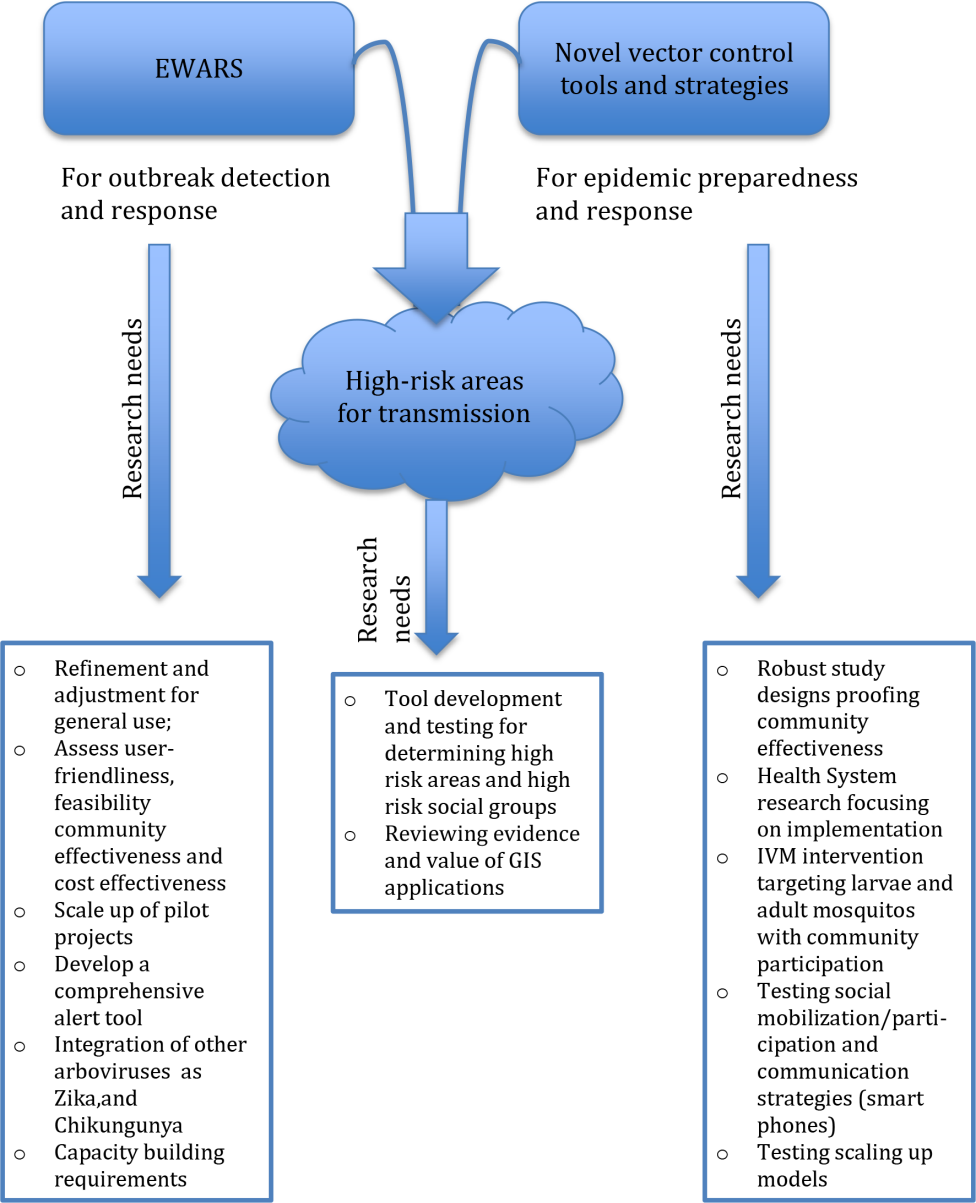
Priority research areas in the EWARS and improved vector control programme.

Based on the above-mentioned research findings on (1) outbreak preparedness of dengue epidemics and (2) improved vector control interventions to control *Aedes*–borne disease transmission, we identified the complementary relationship of the two components. With this in mind we aim to develop a comprehensive intervention package including a) tools for use during the inter-epidemic period—including window screening and community-based campaigns and b) additional interventions for stepwise outbreak response following timely alerts of defined levels. This will require further research (Fig 3).

## DENV/CHIKV/ZIKA transmission control

### Further research needs for contingency planning and the Early Warning and Response System (EWARS)

The indicator-based early warning system for dengue outbreaks and the translation of the technical handbook into national policy requires further evaluation and development in the following areas:

- Implementation at the appropriate level of the health system to assess needs, user-friendliness, feasibility of application, and training requirements;
- Refinement and adjustment for general use;
- Evaluation of the effectiveness at impacting transmission via early response and cost effectiveness in support of country adoption;
- Extension of the EWARS to include additional alarm indicators, particularly qualitative variables, in an alert algorithm, using decision trees and variable weighting;
- Integration of Zika and chikungunya into the alert tool as historic data accumulate;
- Scaling up implementation in current and new countries;
- Strengthening in-country capacity and ensuring sustainability.

Additional research will be required for adapting the alert algorithm after implementation of new interventions, such as a dengue vaccine—preferably in combination with vector control activities—and/or *Wolbachia*-infected mosquitoes.

### Further research needs identified for scaling up vector interventions

The optimal strategy to deliver vector control tools needs to take into account the principles and key elements of Integrated Vector Management (IVM), which include evidence-based decision making, judicious use of insecticides, and social mobilization and collaboration within the health sector and beyond [36]. Such a strategy requires further research, with the following specific needs:

- Robust study designs to produce evidence of effectiveness against transmission.
- Health system research focusing on implementation issues.
- Complex intervention strategies targeting larvae and adult mosquitos as part of community. based-campaigns, including delivery of several interventions from an operational perspective.

More specifically, we also need to:

- Identify the best practices and strategies for a successful partnership model, including social (community) participation.
- Analyse the enabling and the limiting factors for successful inter-sectoral work at the municipality level.
- Develop and evaluate effective and cost-effective methods to promote social participation in urban environments, using modern communication technologies.
- Identify decision makers’ perceptions and biases on the risks, costs, and benefits of improved interventions.
- Develop a framework that facilitates decision-making regarding how successful small-scale pilot research projects should be taken to scale.

### Research needs for defining the priority areas for the application of improved vector control interventions during the inter-epidemic period and outbreaks

DENV, ZIKV and CHIKV transmission mainly occur in urban and periurban areas—usually consisting of large and heterogeneous districts. National programme managers, district health, and control programme staff urgently require a tool that identifies priority areas for action, particularly where the interventions should start, and ideally includes a rapid diagnostic test to distinguish between the different *Aedes*-borne diseases. In the presence of predictive outbreak alarms, a method to focus interventions spatially in a targeted fashion would likely increase the efficiency of available resources. Indeed, the absence of such a tool is a major concern often expressed by national programme managers; after receiving an alarm signal at the district health office, staff need clear guidance on where to intervene with early response actions, particularly for highly focal vector control measures such as fogging.

A Geographical Information System (GIS) risk mapping tool that includes appropriate evidence-based variables such as vector densities, historical clustering of cases, and/or population movements (yet to be developed and tested) could potentially overcome these difficulties. Research needs include:

- Developing and pilot testing using GIS software;
- Field evaluation of the application in high-risk areas;
- Feasibility, cost, and acceptance studies;
- Integration and combined use of the GIS application with EWARS.

## From research to policy support

Following the research-to-policy forum, evidence supporting policy uptake of interventions were extracted with a view of informing guidelines and policy recommendations, including the process being undertaken by WHO to update the 2009 *Global Dengue Guide*. Specific considerations regarding the translation of research findings into policy were as follows:

### Research on outbreak detection and response (EWARS) and contingency planning: Implications for policy

The technical handbook for contingency planning, along with the operational guide describing the steps of establishing the EWARS at national, state, or even district level are being considered by several ministries of health. The first year of implementation, monitoring, and evaluation will be crucial to understanding the benefits and limitations of this system. The feedback will influence the direction of the EWARS, whether it can be recommended for general use or whether further adaptations are necessary.

Vector surveillance data (generally larval/pupal surveys) are frequently captured using non-standardised methods. These data are often used for monitoring the success of an intervention rather than generating time-series data to monitor fluctuations in vector abundance. Furthermore, adult mosquito data are not routinely captured and less so investigated for the presence/absence of viruses. Together, these factors have limited the predictive ability of vector dynamics for epidemic transmission and contribute to the lack of evidence of a correlation between vector density and disease incidence. A transition to capturing adult mosquito data may be a better option. Moreover, establishing the number of adult mosquitos positive for virus will enable the production of fine spatial scale risk maps for targeting of interventions.

### Research on improved vector management approaches: Implications for policy

Complex intervention strategies targeting larvae and adults as part of community-based campaigns are effective, although it is difficult to disentangle the contribution of each individual component. It is now important to assess how these interventions can be best delivered and evaluate their impact from an operational perspective. Also necessary are studies that can tease apart the individual impacts of interventions that comprise complex community-based campaigns.

According to the available data on successes of house screening in reducing indoor and outdoor vector densities—as well as some evidence to suggest an impact on transmission—house screening seems to be a promising measure to limit the transmission of *Aedes*-borne arboviral infections, provided suitable house structure, coverage, acceptability, and sustainability. Additionally, evidence exists in support of complex community-based campaigns to reduce the circulating *Aedes* population suggesting that these can directly translate into an impact on disease transmission, although further studies are required.

### Research on risk mapping tool: Implications for policy

A shift away from global risk maps (which show the burden of dengue and other *Aedes*-borne diseases in countries and regions) towards fine-scale risk maps (which visualize, for operational purposes, the infection risk at sub-district level called “localities” or “health areas” or “PHC areas,” etc.) is required to better focus vector control interventions. The addition of fine-scale variables, such as adult mosquito abundance and entomo-virological parameters, will facilitate this process. Human movement and contact-tracing studies are also crucial to understand the role of human behaviour in the transmission of *Aedes*-borne diseases (see also Vazquez-Prokopec (2017) [37]). Finally, studying the impact upon prevailing herd immunity of newly introduced vaccines becomes now a necessary addition. Together, such advancements will enable national control services to focus and prioritise areas for intervention, both in-between and during epidemic periods.

## Conclusions

Research into the three above-mentioned areas is essential. The combination of early warning systems, improved vector control, and spatial mapping is expected to increase substantially the efficiency of the limited resources countries can afford. Further research is required to bring these three areas together, some of which is already underway. The use of both community-based and public-sector approaches will empower populations at risk, reduce the per-capita cost of interventions, build sustainability, and increase the impact of vector control interventions.

Dengue control traditionally consumes a substantial proportion of the generally limited human and financial means of a large number of countries yet often fails to curb epidemics early enough. In addition, Zika and chikungunya epidemics are now occurring in dengue-endemic areas, further burdening already overstretched health systems. Therefore, more investments are needed into research to identify what really works and how—and should thus be implemented—and what does not, and is a waste of resources.
